# Influence of Scanning Strategies on Processing of Aluminum Alloy EN AW 2618 Using Selective Laser Melting

**DOI:** 10.3390/ma11020298

**Published:** 2018-02-14

**Authors:** Daniel Koutny, David Palousek, Libor Pantelejev, Christian Hoeller, Rudolf Pichler, Lukas Tesicky, Jozef Kaiser

**Affiliations:** 1 Institute of Machine and Industrial Design, Faculty of Mechanical Engineering, Brno University of Technology, 616 69 Brno, Czech Republic; palousek@fme.vutbr.cz (D.P.); 153284@vutbr.cz (L.T.); 2 Institute of Materials Science and Engineering, Faculty of Mechanical Engineering, Brno University of Technology, 616 69 Brno, Czech Republic; pantelejev@fme.vutbr.cz; 3 Institute of Production Engineering, Faculty of Mechanical Engineering and Economic Sciences, Graz University of Technology, 8010 Graz, Austria; christian.hoeller@tugraz.at (C.H.); rudolf.pichler@tugraz.at (R.P.); 4 Institute of Physical Engineering, Faculty of Mechanical Engineering, Brno University of Technology, 616 69 Brno, Czech Republic; kaiser@fme.vutbr.cz

**Keywords:** selective laser melting, aluminum alloy, EN AW 2618, scanning strategy, relative density, mechanical properties

## Abstract

This paper deals with various selective laser melting (SLM) processing strategies for aluminum 2618 powder in order to get material densities and properties close to conventionally-produced, high-strength 2618 alloy. To evaluate the influence of laser scanning strategies on the resulting porosity and mechanical properties a row of experiments was done. Three types of samples were used: single-track welds, bulk samples and samples for tensile testing. Single-track welds were used to find the appropriate processing parameters for achieving continuous and well-shaped welds. The bulk samples were built with different scanning strategies with the aim of reaching a low relative porosity of the material. The combination of the chessboard strategy with a 2 × 2 mm field size fabricated with an out-in spiral order was found to eliminate a major lack of fusion defects. However, small cracks in the material structure were found over the complete range of tested parameters. The decisive criteria was the elimination of small cracks that drastically reduced mechanical properties. Reduction of the thermal gradient using support structures or fabrication under elevated temperatures shows a promising approach to eliminating the cracks. Mechanical properties of samples produced by SLM were compared with the properties of extruded material. The results showed that the SLM-processed 2618 alloy could only reach one half of the yield strength and tensile strength of extruded material. This is mainly due to the occurrence of small cracks in the structure of the built material.

## 1. Introduction

Selective laser melting (SLM) is a progressive method of additive manufacturing, mainly used for the rapid production of prototypes and lightweight components with complex geometry. For the latter, alloys with a good strength-to-weight ratio, like high strength aluminum (series 2000 and 7000) are best suited [1]. These alloys are usually considered difficult to weld. Due to its ability to maintain mechanical properties under temperatures of up to 300 °C the aluminum alloy EN AW 2618 is typically used in the automotive and aerospace industry, for pistons, turbochargers and rotary components of aircraft engines [2].

Nowadays, of all aluminum alloys, those with Al-Si base are predominantly used for the SLM process. These alloys, originally used for casting, are mainly used because they are relatively easy to work due to the small difference between the melting and solidification temperature. From these alloys, AlSi10Mg and AlSi12 were intensively studied [3,4,5,6,7,8]. The mechanical properties of these materials in the SLM state were investigated [9,10,11,12] and high cycle fatigue behavior was observed [13,14,15,16]. Compared to the cast material, these alloys in the SLM state reached higher mechanical properties, but lower fatigue properties.

On the other hand, the processing of Al-Cu alloys by SLM technology is difficult, as is shown by initial studies of these alloys [17,18,19,20]. Karg et al. [17] studied the processing of EN AW 2022 and EN AW 2024. The approach for finding optimal processing parameters went from single-track welds through wall tests to cube sample testing. A SLM 50 machine from Realizer GmbH with maximum laser power (LP) of 100 W was used for these experiments. The layer thickness was kept at a constant value of 30 µm. A relative density above 99.5% was achieved with LP 100 W and laser speeds (LS) between 150–300 mm/s for alloy EN AW 2024. Alloy EN AW 2022 showed a narrower processing window with LS between 265–295 mm/s.

Ahuja et al. [18] described the processing of alloys EN AW 2219 and EN AW 2618. They used the same approach and SLM machine as Karg et al. [17]. The best achieved relative density was 99.96%. Authors from both studies observed an increase in relative density using support structures for the fabrication of small cube samples. According to the author, this is mainly due to the reduction of heat transfer between samples and the building platform when the cross-section area of support is rather small compared to the face of the cube samples. They also observed that relative density is not in direct correlation with volumetric energy density.

The authors Zhang et al. [19] arrived at other results with alloy EN AW 2024, as they observed a critical volumetric energy density (ED) of 340 J/mm^3^. All samples with an energy density higher than this value showed a relative density above 99.5%. To achieve such high energy input, the laser speed had to be lowered to values of around 80 mm/s, while a laser power of 200 W was used. A tensile test and hardness measurement was performed on SLM samples in their as-built state. Results showed an increase in yield strength (YS) from 75 MPa (as-cast state), to 276 MPa (SLM state). Ultimate tensile strength (UTS) increased from 185 MPa to 402 MPa and hardness form 80 HV0.2 to 111 HV0.2.

Several studies have focused on the influence of scanning strategies during the processing of different materials. Thijs et al. [8] found a relation between the crystallographic texture of AlSi10Mg alloy and a applied scanning strategy as a consequence of directional solidification due to the moving heat source, while Read et al. [11] found the island size to have the least influence on porosity formation in comparison to laser speed and laser power when processing AlSi10Mg alloy using the island scanning strategy. In addition, Lu et al. [21] studied the mechanical properties and residual stress induced in the Inconel 718 alloy while using a different size of island scanning strategy. They found that the 5 × 5 mm island size is promising for lowering the residual stress. They observed the cracks on the border of the islands, thus the enlargement of island size (lowering the number of borders) produced material with lower porosity and higher elongation. On the contrary, the smaller island produced material with slightly higher UTS and lower residual stress. Carter et al. [22] also investigated the island scanning strategy. They found the influence on the grain structure of nickel superalloy CM247LC by the localization of cracks in the border zones of the islands. They estimated that the higher crack occurrence is present at the high-angle grain boundaries. Popovich et al. [23] showed that the different process parameters together with the scanning strategy strongly affects grain orientation and the resulting mechanical properties of Inconel 718, thus functionally graded materials can be produced with this approach.

This paper builds mainly on the findings of the initial study of high power processing of EN AW 2618 proposed in two articles, Koutny et al. [24] and Koukal et al. [25]. In these studies, a SLM 280^HL^ machine from SLM Solutions with a maximum laser power of 400 W was used. Experiments comprised of single-track welds and volume samples (cubes 5 × 5 × 5 mm), all with a layer thickness of 50 µm. A wide range of processing parameters (laser power, laser speed, and hatch distance) was studied. For cube tests, a relative density above 99% was achieved with LP 200 W, LS 200 mm/s and a hatch distance (HD) of 110 µm. These results correspond with the other studies of Al-Cu alloy mentioned above. However, low surface roughness was observed with parameters LP 400 W and LS 1400 mm/s. In all of the above mentioned articles focused on aluminum alloy EN AW 2618 [17,18,20], the authors describe the presence of a large number of cracks in the samples.

The aim of this study is a detailed examination of the process parameter window found in previous studies [24,25]. Larger cube samples which have been built and evaluated to explore the influence of different scanning strategies and other SLM process parameters on relative density and mechanical properties, have not yet been investigated.

## 2. Materials and Methods

### 2.1. Powder Characterization

The metal powder used in all experiments was fabricated by an inert gas atomization process and supplied by TLS Technik GmbH. The particle distribution specified by the vendor was 20–63 µm. The powder was not additionally sieved and was used as received from the vendor. Several analyses were made of the powder samples.

A chemical analysis was made with the inductively coupled plasma-optical emission spectrometer iCAP 6500 ICP-OES (Thermo Fisher Scientific, Cambridge, UK); the results are given in Table 1. The chemical composition of the analyzed powder corresponds to the EN 573-3 standard [26] of Al-Cu alloy EN AW 2618.

**Figure 1:**
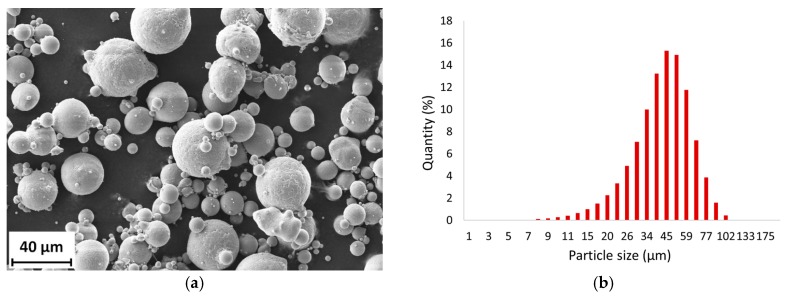
SLM powder characteristics; (**a**) particle shape (SEM); (**b**) particle size distribution chart.

To determine the morphology of particles a scanning electron microscopy (SEM) analysis using the Zeiss Ultra-Plus 50 analytical system was carried out. It can be seen that most particles have a spherical shape satisfactory for SLM processing (Figure 1a). The powder size distribution was measured by laser particle size analyzer Horriba LA-960. The results can be seen in the distribution chart (Figure 1b). The powder meets the distribution specified by the supplier. The particle mean size is 40.1 µm and median size is 39.1 µm. A total of 90% of particles have a size between 22–59 µm, therefore a layer thickness of 50 µm was used.

### 2.2. Fabrication of Samples

All samples were fabricated on the SLM 280^HL^ machine (SLM Solutions Group AG, Lubeck, Germany) equipped with a 400 W ytterbium fiber laser YLR-400-WC-Y11 (IPG Photonics, Oxford, MA, USA) working with continuous wave. The laser beam was focused to a diameter of 82 µm and had a gaussian shape. Nitrogen was used as the protective atmosphere. The overpressure in the building chamber during the build process was 10–12 mbar and the oxygen level was kept below 0.2%. The temperature of the building platform was 80 °C.

To achieve the best mechanical properties of EN AW 2618 in the SLM state, several processing strategies were examined. The evaluation process consisted of three steps:

1. Single-track welds
2. Volumetric cube samples
  - Meander strategy
  - Chessboard strategy
  - Hull and core strategy
  - Pre-sintering strategy
  - Samples on support structures
  - Samples with high platform heating
3. Samples for tensile testing

#### 2.2.1. Single Track Welds

The aim of single-track welds is to find the areas with a suitable combination of main process parameters that would be prospective for the building of low porosity volumetric samples. Because of non-consistent results in the initial study [24], single-track experiments were performed again for better accuracy. To ensure uniformity of the coated powder layer, a manual recoating device was used. Images of individual tracks from the top view were made for the visual evaluation of the continuity and uniformity of the weld tracks. In the case of a bumpy surface or balling effect, the evaluation of the track was penalized. A cross-section of samples was also analyzed and measurements of the main dimensions were made. The main evaluation was based on a weld height (*h*) to weld width (*w*) ratio combined with height above substrate (*a*) to weld height. The symmetrical welds (*h*/*w* ~ 1) with a symmetrical position on the substrate (*a*/*h* ~ 0.5) and optimal height above material (*a*/50 > 0.5) were preferred. A wide range of process parameters was evaluated. LP varied in range from 100 W to 400 W in 50 W steps and LS from 50 mm/s to 1700 mm/s in 50 mm/s steps. Tracks with a lower energy input (LP below 300 W and LS above 1200 mm/s) were excluded from experiments.

#### 2.2.2. Volumetric Cube Samples

**Figure 2:**
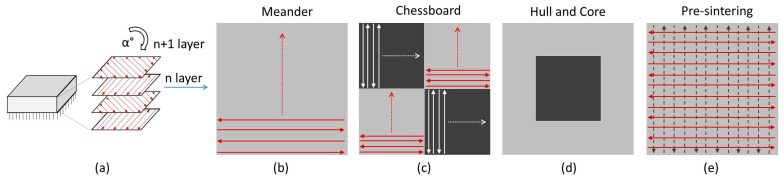
Graphical representation of used strategies; (**a**) layer-based approach, with rotation of scan pattern in follow up layers; (**b**) Meander strategy; (**c**) Chessboard strategy; (**d**) Hull and core strategy; (**e**) Pre sintering strategy.

The general approach of SLM is layer-based (Figure 2a) and regularly the rotation of scanning patterns between layers is used to minimize material porosity. Thus, for all experiments with volumetric samples, a rotation angle of 73° was used.

The meander strategy is the basic strategy generally used for hatching. Neighboring vectors of the laser path are scanned with a constant hatching distance in the opposite direction over the entire layer. The scanning of larger areas with a meander strategy could induce higher residual stresses due to the high thermal difference in the opposite ends of the scanning vectors (Figure 2b).

Splitting the entire area into numerous local areas combined with a different scanning order is considered beneficial to eliminate stresses and overheating of local areas. This approach is known as island scanning or the chessboard strategy. In this strategy (Figure 2c), a cross-section area of the sample is dived to several sub-areas, where each sub-area (one square of a chessboard) is scanned with the meander strategy. The hatching angle between “black” and “white” fields is 90° (Figure 2c).

In the case that different process parameters within a single layer are beneficial for the component processing, the hull and core strategy can be used. In this strategy the sample is divided into two areas. On the sample’s outer contour, there is the hull area and in the center of the sample is the core area. For each of these areas different contour and hatch parameters can be set. This allows even better distribution of energy in one layer of the sample.

Also, a double layer exposure can be used to eliminate defects. This approach is referred to as re-melting or pre-sintering, according to the energy used in the first exposure. Since the effect of re-melting has already been described earlier in a separate article [27], the effect of pre-sintering is studied. The first exposure is treated with lower laser power to preheat the layer while maintaining a uniform powder height. It is expected that it would be beneficial for the situation during a second exposure with full power, where this could result in lower temperature gradients, and energy entering the material could be higher while maintaining the same scanning speed.

The samples were mechanically ground on emery paper 1–2 mm bellow the top surface. If necessary, even polished with 3 µm and 1 µm diamond slurry. Fuss reagent was used as an etchant to differentiate grains and phases. Images of the polished surface were captured by the OLYMPUS SZX7 (Olympus Corporation, Tokyo, Japan). Images were then analyzed in software ImageJ (1.51 g, National Institutes of Health, MD, USA), where a threshold function was used to determine the relative porosity of samples.

#### 2.2.3. Tensile Testing

The combination of parameters evaluated as the best for each of the tested processing strategies was used for the fabrication of larger samples for the testing of mechanical properties. SLM billets of prismatic shape with a square cross section (13 × 13 mm) and length of 83 mm were fabricated. The axes of SLM billets were parallel with the plane of the building platform, i.e., the loading axis during the tensile test was perpendicular to the building direction. Cylindrical testing samples with a nominal diameter of 8 mm and gauge length of 40 mm (according to DIN 50125) were machined out of the SLM processed material.

Tensile tests were made on a Zwick Z250 testing machine with a loading speed 2 mm/s at room temperature. For hardness measurement the HV 0.3 device LECO LM 274 AT (LECO Corporation, Saint Joseph, MI, USA) was used. For each tested strategy a set of three samples was fabricated, machined and tested. Average values and range of yield strength (YS) and ultimate tensile strength (UTS) were evaluated unless otherwise specified.

For comparison, tensile tests of standard wrought material (supplier Strojmetal Aluminium Forging s.r.o., Kamenice, Czech Republic) in states without heat treatment and with heat treatment T6 were performed.

## 3. Results and Discussion

### 3.1. Single Track Welds

**Figure 3:**
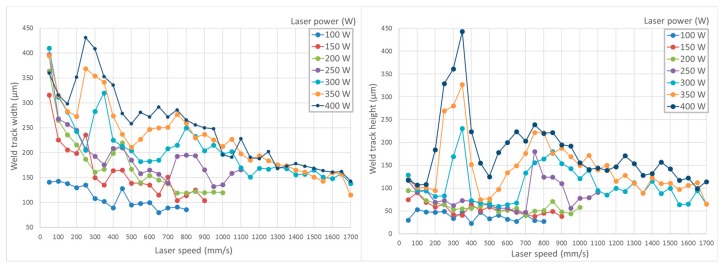
Single track weld evaluation, width and height of the weld according to applied LS and LP.

**Figure 4:**
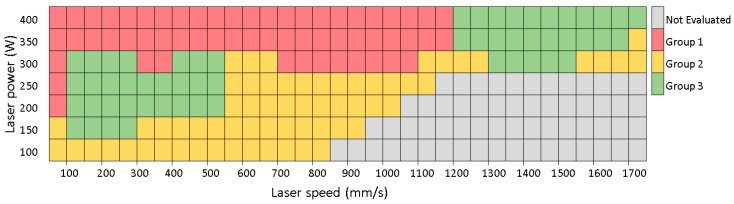
Process window identified from single weld track evaluation.

**Figure 5:**
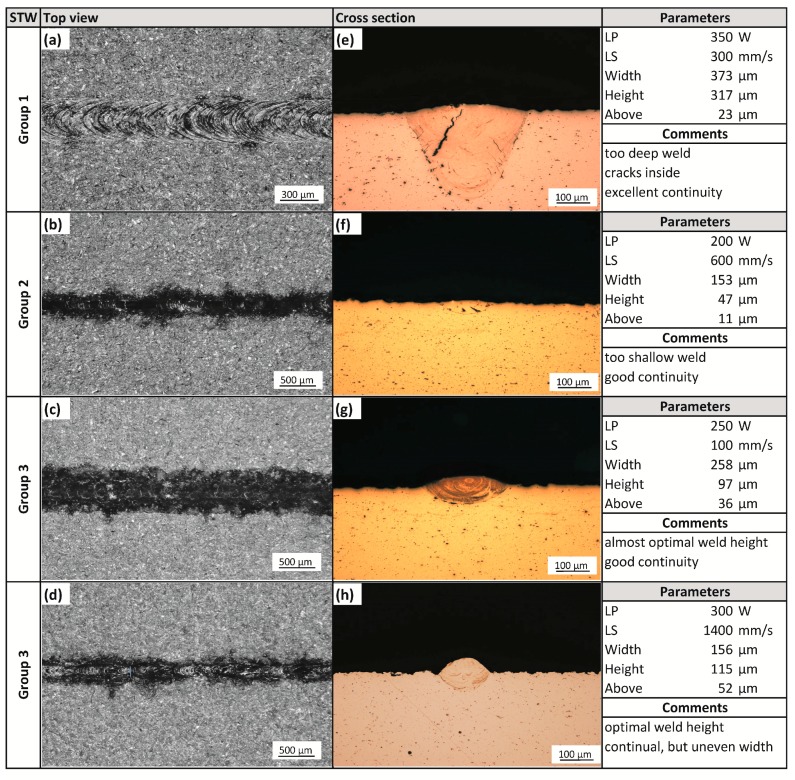
Representative samples of single track weld for each group of welds; (**a**–**d**) top view; (**e**–**h**) cross section.

Single-track welds were used for the evaluation of a wide range of processing parameters (Figure 3). Because there were only two laser-related parameter variables, laser power and laser speed, the influence of other parameters was excluded or minimized. Weld tracks were sorted into three groups according to the chosen criteria (Figure 4). Group 1 welds are characterized as too deep and prone to cracking. In advance, deep welds can easily cause keyhole pores. Thus, this type of weld is not considered optimal for the fabrication of volumetric samples with low porosity. Group 2 welds are considered as too wide, with low depth and low height above the substrate that could result in being too large and often re-melting. Group 3 welds are those with a good height to width ratio suitable for producing low porosity material. Figure 5 is complementary to Figure 4 and shows the representative samples of weld tracks for each group of welds with top view, cross section, measured parameters and evaluation comments. Two promising processing windows with optimal shape of the track were found. The first one is in the area of lower LS (100–400 mm/s) and LP (200–250 W). The second one is in the area of higher LS (1200–1500 mm/s) and higher LP (350–400 W). These results correspond to the results of the cube samples within the initial study [24,25]. While in studies [17,19] the optimal values for aluminum alloys 2219 and 2024 were found only in the range of low scanning speeds, below 200 mm/s.

Minimization of the influence of other parameters has certain advantages and disadvantages. From the overview of weld track proportions and continuity, the process windows were found. Thus, the hatch distance could be set according to the desired overlap of the weld tracks. The optimum overlapping was considered as 60% which resulted in the use of a 110 µm hatch distance for the Group 3 welds with lower speeds and a 65 µm hatch distance for the Group 3 welds with lower speeds.

The disadvantage of the single-track weld evaluation is that for multiple tracks, arranged side-by-side, the thermal situation may be different. E.g., the measured parameters of the weld tracks may be changed due to a higher temperature during the build of volumetric samples.

A rapid change in the depth of the melt pool was observed (Figure 3) within a higher laser power (300–400 W) and laser speed between 200–400 mm/s. This may be due to a change in the absorptivity of the material [28], where more energy is absorbed by the melt pool within this area of the process parameters.

This behavior during processing is undesirable and in connection with the rise of the temperature during fabrication of the volumetric samples could cause a major shift of the optimal processing window far from the expected values.

### 3.2. Meander Strategy

**Figure 6:**
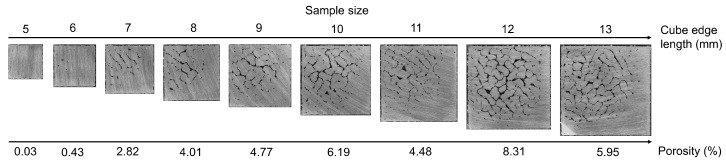
Metallography of different size cube samples fabricated using the meander strategy, top view of samples cross section (13 mm sample also shows the starting side and direction of scanning vectors in follow up layers, this was identical for all sample sizes) with relative density evaluated from the cross sections.

A wide range of processing parameters, with use of the meander strategy, were examined within the initial study [26]. The results showed a relative porosity of over 99% for the samples with scanning speeds of 200 mm/s and laser power 200 W. However, only small cube samples (5 × 5 × 5 mm) with the meander strategy were examined. Therefore, the aim of the first volumetric test was to evaluate the influence of sample size on porosity. In this test, cube edge length varied from 5 mm to 13 mm. All samples within this test were fabricated with the same process parameters, and those with the best result in the initial study [24] were used (LP = 200 W, LS = 200 mm/s and HD = 110 µm). The porosity of samples rapidly increased with increasing cube sizes (Figure 6).

This implies that the temperature distribution during the sample fabrication changes. With elongation of the specimen edge, the time between the scanning of the neighbouring track is increased and the temperature drop of the track is higher, thus the supplied energy is probably insufficient in comparison to the small sample. It is apparent that the meander strategy is not optimal for samples with a larger volume. According to this finding, all following experiments were made with a cube size of 13 × 13 × 5 mm. Mostly, irregular shaped pores, probably induced by hot cracking, were observed (Figure 6). However, the area that was scanned first was without pores.

### 3.3. Chessboard Strategy

The influence of several process parameters within the chessboard strategy has been investigated. Firstly, the standard setup of the chessboard was used with LP variation between 190 and 200 W while LS varied in the range of 100–300 mm/s. Secondly, the scanning order of individual fields was changed, and lastly the influence of the chessboard field size, which varied from 5 × 5 to 1 × 1 mm, was evaluated.

**Figure 7:**
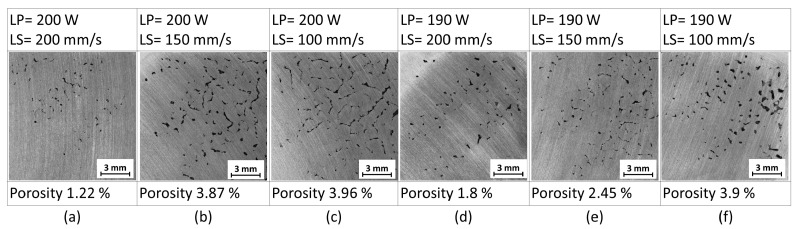
Influence of LP and LS variance on the defect formation using the chessboard strategy.

Results of the first test show bands with a high number of defects alternating with bands without a major occurrence of defects (Figure 7a–f). From that behavior, there was obvious dependence on the chessboard scanning strategy. The lowest values of porosity were achieved with the LP = 200 W and LS = 200 mm/s.

**Figure 8:**
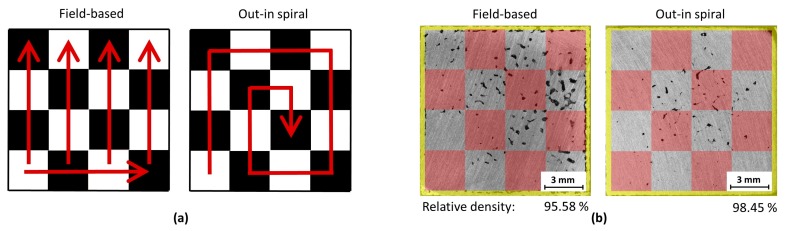
(**a**) Scanning order of individual chessboard fields for different settings of the chessboard strategy; (**b**) Comparison of sample defects for field-based and out-in spiral setting of the chessboard strategy; to visualize the real in size and distribution of black and white fields over the ground sample, the white fields are shown with a transparent red color.

The presence of alternating bands of defects can be explained by the field-based scanning order of the chessboard strategy (Figure 8a). With the field-based setting, fields are scanned from the bottom-left corner to the top-right corner. The “white” fields are scanned in the first run over the actual layer, and then the black fields are scanned in the second run over the actual layer. This scanning order of white and black fields is used in all cases of the variants of the chessboard strategy. With the setting out-in the spiral, fields are scanned from the bottom-left corner to the center of the sample.

The influence of different scanning order settings on the porosity value and defect distribution is shown in Figure 8b. In general, the measured porosity of samples with the field-based setting was 2% greater than out-in spiral setting and up to 75% of irregular shaped pores in the sample were located within the “black” fields, therefore in the area that is scanned last. With the out-in spiral setting, the irregular pores were smaller in size and located near the center of the sample, again the area which is scanned last.

**Figure 9:**
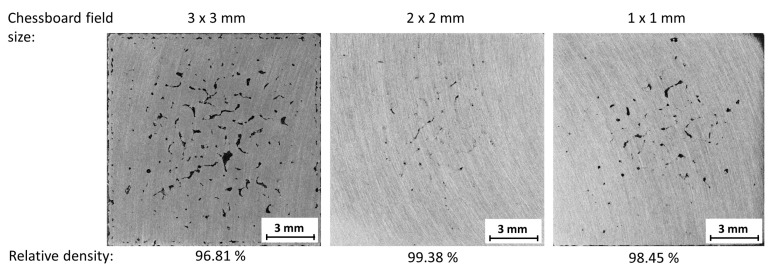
Influence of chessboard field size on the relative porosity.

With the chosen out in spiral scanning order, the last part of the test was conducted. It was expected that with a smaller chessboard field size a better heat distribution over the layer could be achieved, which could be beneficial to minimize the amount of defects. The lowest porosity values were reached with a field size of 2 mm, while the most defects were localized in the center of the sample (Figure 9).

### 3.4. Hull and Core Strategy

Because the results of the chessboard field size test (Figure 8b) showed an area of about 3 mm in thickness around the sample without major defects, for the test the hull thickness was set to 3 mm. Thus, the aim was to preserve these conditions in the hull area and optimize the core area parameters only. The hull parameters remained the same as in the best chessboard experiments (LP = 200 W and LS = 200 mm/s). The laser speed for the core area was set at constant value of 100 mm/s. This was due to the known poor results for 200 mm/s and the prospective porosity values achieved in previous tests at lower speeds. The LP varied from 100 W to 400 W.

**Figure 10:**
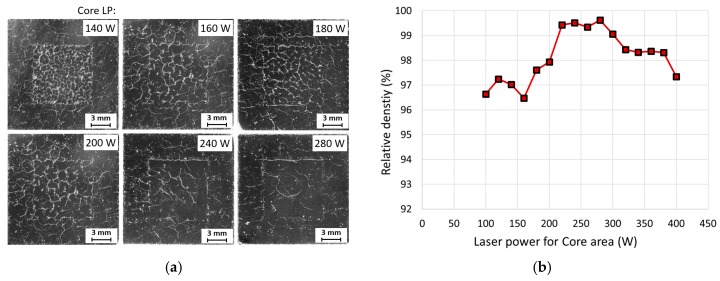
Defects in cube samples fabricated using the hull and core strategy; (**a**) metallography, top view of sample’s cross section; (**b**) dependence of relative density and laser power of the core area.

As can be seen from Figure 10, the best result was achieved with LP = 280 W. Samples below this value showed hot cracking defects and above it showed increased gas pores. The relative density for the best achieved sample was 99.62%. However, these samples showed crack occurrence mostly on the border between the hull and core area (Figure 5a).

### 3.5. Pre-Sintering Strategy

**Figure 11:**
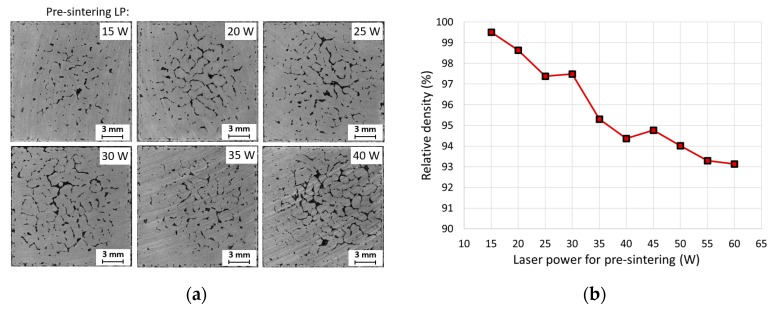
Defects in cube samples fabricated by pre-sintering strategy; (**a**) metallography, top view of sample’s cross section; (**b**) dependence of relative density and laser power.

For AlSi10Mg alloy, Aboulkhair et al. [5] achieved best results of relative density with the pre-sintering strategy, therefore the pre-sintering strategy was chosen to explore if there is a positive effect on the relative density of EN AW 2618 alloy. Within this strategy, every layer is scanned twice before the next layer of powder is applied. The first scanning (pre-sintering) had a lower laser power setting, thus the powder was only sintered. The second scanning (melting) had standard parameters and thus the powder was fully melted. Ten samples with melting parameters of LP = 200 W and LS = 200 mm/s with the chessboard 2 × 2 mm strategy were fabricated. For pre-sintering, LP varied from 15 W up to 60 W, while a scanning strategy and laser speed identical to the melting was used. Figure 11 shows that with increasing pre-sintering laser power, the porosity of samples increased. Samples without pre-sintering achieved better relative density, therefore pre-sintering has a negative impact on the quality of samples.

### 3.6. Influence of Support Structures

Ahuja et al. [18] and Karg et al. [17] both observed an increase in the relative density of samples that were built on support structures compared to those that were built directly on a build platform. In the initial study for EN AW 2618 [24,25] this influence was examined, but no change in the sample’s quality was observed. However, for larger cube samples this influence has not yet been evaluated.

Support structures were created in the Materialise Magics software (21.11, Materialise nv, Leuven, Belgium). The type of support used was block support. Within the first test, the geometry of support structures was optimized. The best results were observed for hatching (distance between two lines of block support) 0.9 mm. To increase the toughness of support structures 5 cone supports were added, four to the corners and one to the center of sample.

**Figure 12:**
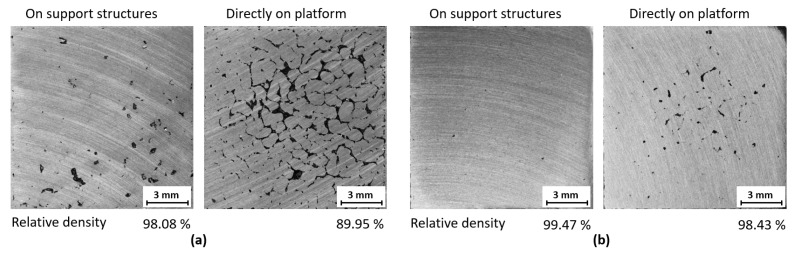
Influence of support structure; (**a**) Meander strategy; (**b**) Chessboard strategy.

Samples with the meander and the chessboard strategy were fabricated to compare them with samples fabricated directly on the build platform. Results showed a decrease in porosity for both scanning strategies. For the meander strategy porosity decreased on average by 6% (Figure 12a), for the chessboard strategy the reduction was not so significant. On average by 1.5% (Figure 12b). The decrease in defects was probably caused by the lower temperature gradient. The support structures act as isolation of the processed material from the built platform because it has lower heat capacity than a much larger solid block represented by the built platform. Heat transfer is then much slower.

### 3.7. Samples with Higher Platform Heating

The main goal of this experiment was to heat up the platform to higher temperatures in order to avoid the occurrence of cracks in the sample due to the reduced temperature gradient between the sample and the platform and reduced heat dissipation from the sample. To achieve higher temperatures of the build platform, a high temperature unit (SLM Solutions Group AG, Lubeck, Germany) was used. This unit allows the build platform to be heated up to 550 °C; for this experiment the platform was heated up only to 400 °C to avoid damage of the heating cylinder due to platform expansion at higher temperatures. To withstand this higher temperature of the platform, a ceramic recoating blade came into use instead of a standard silicone one. As for the protective atmosphere, there was a change to the use of argon.

**Figure 13:**
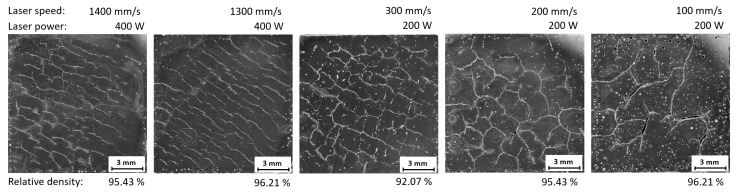
Metallography of samples with higher platform heating and meander strategy.

**Figure 14:**
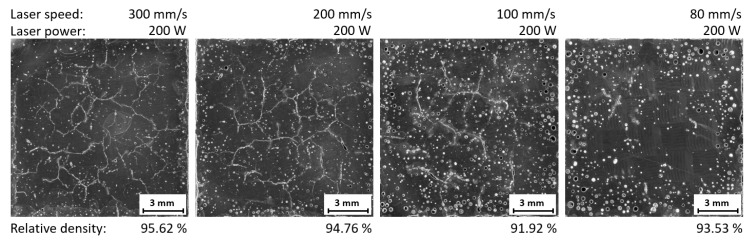
Metallography of samples with higher platform heating and chessboard strategy.

The samples were built on the variation of the two relevant process windows identified in single-track welds (LP = 400 W, LS = 1300–1400 mm/s; LP = 200–300 W, LS = 100–300 mm/s). These variations were done either with the meander strategy or the chessboard strategy and the results can be seen in Figure 13 and Figure 14. Due to the build process, elevated edges could be observed in all samples, but the samples made with the chessboard strategy apparently show a higher trend to these imperfections. In several instances this fact even led to a stop in the processing in order to avoid damage to the ceramic recoating blade. Thus, not all samples were successfully built and analyzed.

In comparison with samples built at a lower platform temperature, an increased incidence of round pores induced by local ablation was observed in all samples. The meander strategy did not show a significant reduction in crack occurrence (see Figure 13). For samples with the chessboard strategy and laser power of 200 W a reduced occurrence of cracks was observed when working alongside decreased laser speed. However, this lower laser speed raised the gas porosity considerably (see Figure 14). The cracks were visible in all samples and no significant change in their quantity was observed; however, a different direction of crack propagation between strategies was observed. For the meander strategy the cracks spread along the diagonal of the sample and for the chessboard strategy cracks spread in all directions.

### 3.8. Mechanical Properties

Fabrication of larger samples for tensile testing was performed after the evaluation of cube samples and the fixation of parameters for each tested strategy. The results of the tensile test are summarized in Table 2. In the case of the meander strategy, the achieved UTS was far below expected values, which prompted the search for other strategies suitable for a more even distribution of heat in the layer. The chessboard strategy showed partial improvement, but the amount of defects was still too high to be comparable with extruded material. The hull and core strategy minimized the large defects, thus the improvement of mechanical properties was expected. However, the cracking on the interface of the hull and core was shown to be the major limiting type of defect. One of the samples was even broken during the machining of the final shape for tensile testing. All samples fabricated directly on the platform exhibited fragile behavior and the yield strength could not be estimated. The pre-sintering strategy showed no improvement in defect minimization, thus the fabrication of larger samples and their tensile testing was not performed.

The best results were achieved for the meander strategy and the samples built on support structures, which is associated with a decrease in the temperature gradient between the previous and fabricated layer. The smaller difference in UTS between the meander and chessboard strategy in the case of the samples built on support structures shows that the effect of the lower temperature gradient between the layers is more significant than the influence of the temperature gradient across the layer.

Unfortunately, the tensile samples under high temperatures were not fabricated due to partial damage of the ceramic blade. To evaluate the influence on the mechanical properties during high temperature processing it is necessary to eliminate the elevated edges in the future.

Generally, material in the SLM state exhibited significantly lower tensile properties in comparison with the extruded state. 

### 3.9. Metallographic Analysis

**Figure 15:**
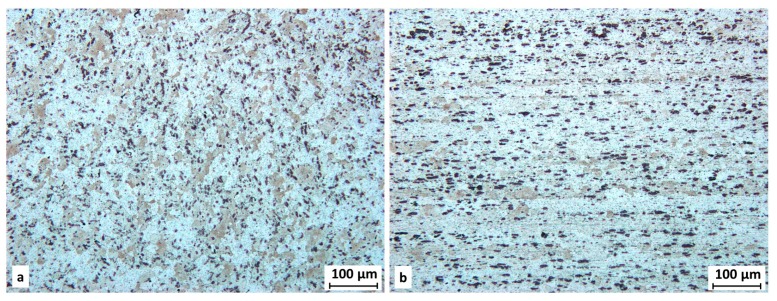
Microstructure of extruded material; (**a**) transverse direction; (**b**) longitudinal direction.

Microstructural analyses of samples prepared from cylindrical specimens after tensile tests (of its threaded heads) were conducted. In Figure 15 the microstructure after the etching of material in the extruded state can be seen. Both the transverse and longitudinal directions are shown (with respect to the cylindrical sample main axis). The preferential orientation of grains and intermediary particles typical for wrought aluminum alloy can be seen. This preferential orientation is connected to the working process of the rod. Intermediary phases of rather a coarse character are present in and out of individual grains.

**Figure 16:**
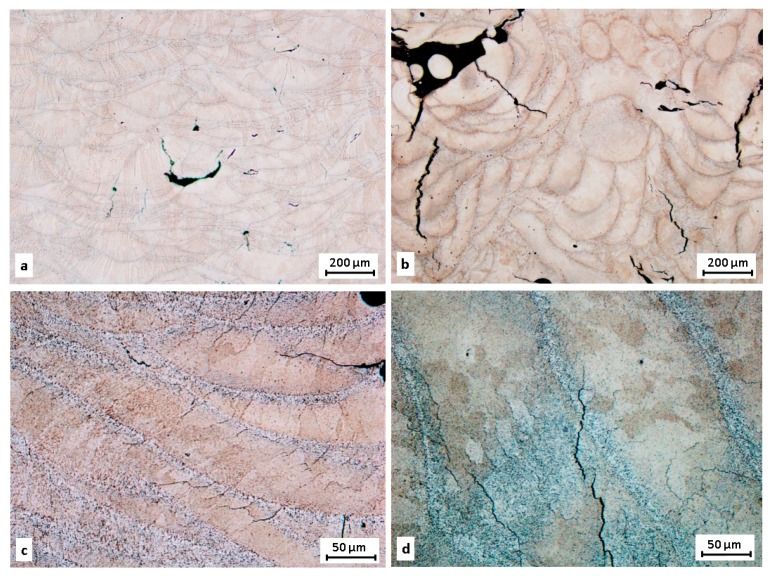
Microstructure of SLM state material fabricated with meander strategy; (**a**,**c**) transverse direction; (**b**,**d**) longitudinal direction.

As expected, the microstructure of SLM-processed material is different (Figure 16a,b). On the samples there is a visible lack of fusion porosity, cracks and gas porosity. At higher magnification (Figure 16c,d) individual weld tracks can be observed. Alongside these tracks intermediary particles can be found. In comparison with the extruded state they are very fine. In this area the initiation of solidification cracks (also known as hot cracking) occurs.

According to Olakanmi et al. [29] the mechanisms behind hot cracking are not entirely understood and there are several theories as to why they occur. One of them is the creation of tensile stress between the solid and liquid phases during the solidification process due to high temperature differences between solid and liquid.

### 3.10. Fractographic Analysis

**Figure 17:**
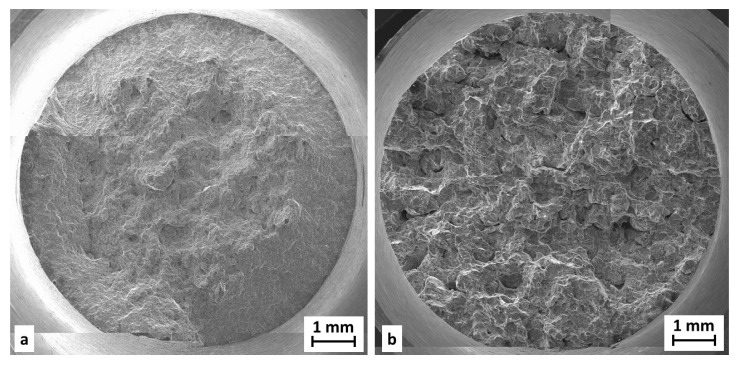
Fraction area of tensile testing samples; (**a**) extruded state material; (**b**) SLM state material fabricated with meander strategy.

Fractographic analysis was performed on samples (both wrought and SLM) after the tensile test. A comparison of the fracture surface of the material in both states can be seen in Figure 17.

**Figure 18:**
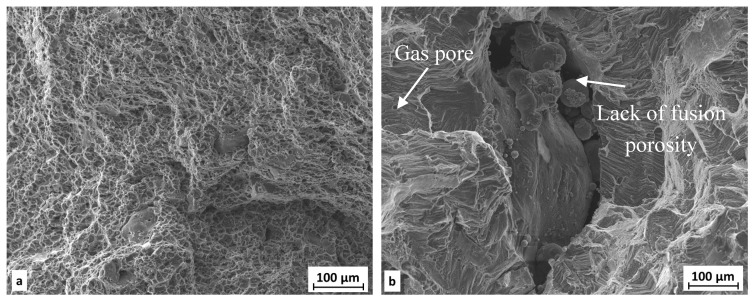
Detail of fraction area—(**a**) extruded state; (**b**) SLM state (meander).

**Figure 19:**
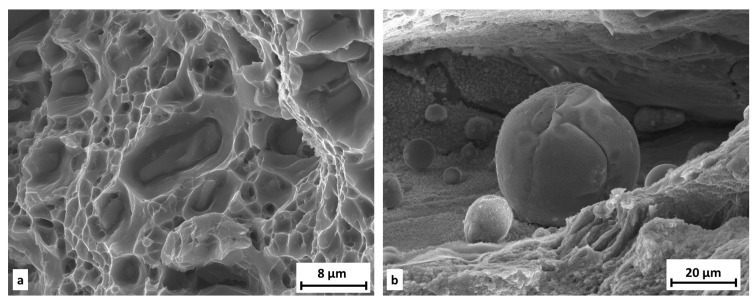
Fraction area of tensile testing samples; (**a**) intermediate particles in dimples; (**b**) unmelted powder in pore.

The fracture surface of extruded material is not as rough as fracture surface of SLM-processed samples. In both states the ductile character of the damage mechanism with dimple morphology was observed. For the SLM state, the dimples are tiny and shallow (Figure 18b), unlike in the extruded state, where the dimples are more pronounced (Figure 18a); moreover intermediate particles in dimples are visible (Figure 19a). For the SLM state, a lack of fusion porosity and gas pores are present on the fracture surface. Inside these defects, unmelted particles of metal powder can be seen (Figure 19b). The surfaces of these pores are covered by an oxide layer.

The results showed some differences in the microstructure of extruded and SLM state material, however hardness measurements for both states are almost identical (100 HV 0.3 for SLM, and 104 HV 0.3 for extruded state). This suggests that the main reason for the different tensile properties is the defects observed in the SLM state. Most of these defects are solidification cracks which were present in samples for all evaluated scanning strategies.

A first experiment with higher platform heating suggests that the reduction of the temperature gradient between the sample and platform may have a positive effect on cracking reduction; however, enormous amount of pores were observed for these samples. A more detailed study with smoother steps between process parameters needs to be performed to fully describe the behavior of crack occurrence with higher processing temperatures.

## 4. Conclusions

It is clear that scanning and processing strategies strongly affect the mechanical properties of SLM-processed aluminium alloy 2618. The best result was achieved with the meander scanning strategy and the sample fabricated on support structures; however, these results are far behind the mechanical properties of the material in the extruded state. The SLM-processed samples reached only half of its values. The main cause is attributed to the formation of solidification cracks, because none of the evaluated scanning strategies resulted in crack-free material. The increase in relative density was achieved by a more even distribution of heat in the fabricated layer using a chessboard strategy. However, it was shown that the use of supporting structures and the reduction of the temperature gradient between the layers had a more significant effect on the mechanical properties. However, a further increase in the base plate temperature to 400 °C did not result in a significant reduction in defects and cracks.

## Figures and Tables

**Table 1:** Chemical composition of the powder.

Elements in wt %							
	Cu	Fe	Mg	Ni	Si	Ti	Al
SLM powder	2.66	1.00	1.39	1.2	0.149	0.206	balance
EN AW-2618 ^1^	1.8–2.7	0.9–1.4	1.2–1.8	0.8–1.4	0.15–0.25	0.2	balance

^1^ according to standard EN 573-3 [26].

**Table 2:** Results of tensile testing.

Tensile Properties	SLM State–Scanning Strategy					Extruded State	
	Meander	Chessboard	Hull and Core	Meander	Chessboard	Heat Treatment	
	Directly on Built Platform			On Support Structures		None	T6
YS (MPa)	-	-	-	177 ± 15	171 ± 17	264 ± 9	365 ± 7
UTS (MPa)	97 ± 10	152 ± 19	48 ± 14	191 ± 15	179 ± 17	383 ± 9	429 ± 7
